# Establishment and Work of a Free Clinic for Children in Montgomery, Ala.

**Published:** 1916-03

**Authors:** G. J. Griel

**Affiliations:** Montgomery, Ala.


### ESTABLISHMENT AND WORK OF A FREE CLINIC FOR CHILDREN IN MONTGOMERY, ALA.

By G. J. Greil, M. D., Montgomery, Ala.

On May 13th, 1914, I requested consent and co-operation of the Montgomery County Medical Society in the establishment of a free clinic or dispensary for children, in the west and or factory district of Montgomery, commenting at the time that it seemed strange to me that free clinics and dispensaries were, as a rule, confined to the larger cities, and to cities that had a medical college and who used same as material and instruction to the student.

The advantage of the dispensary in the cities the size of Montgomery is not recognized as it should be, not only as a benefit to the physician holding such clinic, whereby he is enabled to better familiarize himself with the ordinary and prevalent diseases and their treatment, but it permits him to gather a wealth of material, some of which is out of the ordinary, for study and individual research.

On October 10th, 1914, I made a report to the Society in which I showed the following cases seen and treated to that date:

NUMBER OF CASES

| | | | |
|---|---|---|---|
| Acidosis | 3 | Malaria | 3 |
| Alopecia Areata | 2 | Otitis Media | 3 |
| Anaemia | 1 | Pellagra | 14 |
| Bronchitis | 3 | Periostitis of Rib | 1 |
| Enuresis | 5 | Rheumatism | 3 |
| Feeding | 10 | Rheumatism with Endoc | 1 |
| Furunculosis | 8 | Scarlet Fever | 1 |
| Gastro Enteritis | 1 | Seborrhoea Capitas | 2 |
| Hook Worms | 11 | Syphilis Congenital | 1 |
| Ilio Colitis | 23 | Tuberculosis | 2 |
| Inpetigo | 6 | Tonsilitis | 7 |
| Inguinal Adenitis | 2 | Whooping Cough | 14 |

Whooping cough complicated in several of the above and the remainder minor ailments.

About five hundred prescriptions were dispensed, of which two hundred and ninety-one were paid for by the health department of the city.

Fourteen months have elapsed since the above report. The work has proven so satisfactory, exceeding my hopes and expectations to such an extent, that I feel that other cities should be encouraged to open similar dispensaries and even to extend their work, not only to children but to the different specialties. My dispensary is operated in connection with the West End Neighborhood House, three rooms having been built on a large side porch with doors connecting each room to a large room used as a young men and boys' room by the neighborhood organization for evening gatherings, etc. During the winter months this room is used as a waiting room. These three (3) rooms are used, one for children, one for adults, and the third, a larger room, as a waiting room. Arrangements were made for the surgical and the eye, ear, throat and nose work to be done by the different specialists. The Montgomery Dental Association apportioning the dental work among its members, the patients were sent to their private offices. The laboratory work is done by the city and state laboratorian, and it is prompt and efficient, so that we have as complete a dispensary as could be desired. We can with very little extra trouble be thorough with each case.

The hours for holding the dispensary are from 2 :30 to 3 or 4 on Monday, Wednesday and Friday afternoons, and I make it a point not to let anything interfere with this work, excepting extremely bad weather, absence from the city or emergency work, so that the people of the neighborhood know that if they come they will not be disappointed. I have found that irregular attendance by the physician will soon cause a lack of confidence and a falling off in the attendance.

The following is the routine of the work:

The Superintendent of the Neighborhood House acts in the capacity of nurse, the cases reporting to her, their name, age, etc. In fact, a history of each is taken by her on a blank provided for that purpose. Each case is given a number, the numbers running in succession. The history and physical examination are completed by the physician in charge. The new cases are taken first so as to give plenty of time for a full physical examination and careful diagnosis. Whenever possible a full and comprehensive history is noted. The treatment given forms part of the record. It has been found that twenty to twenty-five minutes is necessary for each new case. The return cases are looked after and treatment continued or changed as the condition requires.

Up to February 1st, 1916, I have received and treated 263 different children, from most of whom I have been able to obtain a fairly good and accurate record. 212 clinics have been held averaging ten patients to each clinic day, making 2120 visits paid to the dispensary. The largest number of patients on any one day was 17, the smallest 3. Some of the children come only the one time for some minor ailment or are referred by the school teacher for our opinion, or are brought by their mothers for a consultation, as it were, and referred back to their family physician. Others with chronic diseases, such as pellagra, endocarditis, etc., are seen at each clinic.

The status of the individual as a dispensary patient is left to the superintendent of the Neighborhood House, as she is as a rule, acquainted with the circumstances of each individual of the neighborhood, and does the visiting, following the cases so far as she can. The dispensary is not able to have a visiting nurse.

Those patients who are able to do so, pay for prescriptions; the others have their drugs furnished them by the city.

From this Dispensary I have been able to report a rather unusual case of amoeba colitis—to collect and study 37 cases of pellagra in children and report my findings, and in conjunction with the State Laboratory to gather and report the results of 665 cases examined for parasitic infection of the intestinal canal and report the results and different parasites found.

That the work has been satisfactory and beneficial I have the assurance of Dr. C. T. Pollard, school physician, and of the teachers of the schools in this district, who have co-operated with me in every way and who have assured me that the attendance at the schools has been much better during the past two years, and that the general condition of the children has been greatly improved.

In conclusion I wish to say that I am thoroughly convinced that dispensaries in smaller cities can be run successfully and will be beneficial to the citizens, the community and the physician in charge.

